# Inter-hospital variation in the utilization of diagnostics and their proportionality in the management of adult community-acquired pneumonia

**DOI:** 10.1186/s41479-018-0059-0

**Published:** 2018-12-25

**Authors:** Stefan M. T. Vestjens, Esther Wittermans, Simone M. C. Spoorenberg, Jan C. Grutters, Charlotte A. van Ruitenbeek, G. Paul Voorn, Willem Jan W. Bos, Ewoudt M. W. van de Garde

**Affiliations:** 1 Department of Internal Medicine, St. Antonius Hospital, Nieuwegein, The Netherlands; 2 Department of Pulmonology, St. Antonius Hospital, Nieuwegein, The Netherlands; 3 Division of Heart and Lungs, University Medical Centre Utrecht, Utrecht, The Netherlands; 4 Department of Pulmonary Medicine, Canisius-Wilhelmina Hospital, Nijmegen, The Netherlands; 5 Department of Medical Microbiology and Immunology, St. Antonius Hospital, Nieuwegein, The Netherlands; 6 Department of Internal Medicine, Leiden University Medical Centre, Leiden, The Netherlands; 7 Division of Pharmacoepidemiology and Clinical Pharmacology, University of Utrecht, Utrecht, The Netherlands; 8 Department of Clinical Pharmacy, St. Antonius Hospital, Nieuwegein, The Netherlands

**Keywords:** Community-acquired pneumonia, Costs and cost analysis, Microbiological testing, Antimicrobial stewardship, Choosing wisely

## Abstract

**Background:**

Utilization of diagnostics and biomarkers are the second largest cost drivers in the management of patients hospitalized with community-acquired pneumonia (CAP). The present study aimed to systematically assess the inter-hospital variation in these cost drivers in relation to antibiotic use in CAP.

**Methods:**

Detailed resource utilization data from 300 patients who participated in a multicenter placebo-controlled trial investigating dexamethasone as adjunctive treatment for community-acquired pneumonia was grouped into 3 categories: clinical chemistry testing, radiological exams, and microbiological testing. Based on the identified top 5 items per category, average costs were calculated per category and per hospital. Antibiotic de-escalation at day 3 and secondary ICU admission were assessed as outcomes for proportionality of diagnostics use.

**Results:**

The mean costs for diagnostics varied between hospitals from 350 (SD 31) to 841 (SD 37) euro per patient (*p* < 0.001). This difference was primarily explained by variation in costs for microbiological testing (mean 195 vs. 726 euro per patient, *p* < 0.001). There was no difference in number of secondary ICU admissions but there was an inverse association between the costs of microbiological testing and level of antibiotic de-escalation. De-escalation occurred most frequently in the hospital with the lowest cost for microbiological testing (48% vs. 30%; *p* = 0.018). The latter hospital had an automated physician alert system in place to consider a timely iv-to-oral switch of antibiotics.

**Conclusions:**

Large inter-hospital variation exists in resource utilization, mainly in microbiological diagnostics in the management of adult patients with community-acquired pneumonia. A counterintuitive inverse association between the magnitude of these costs and the amount of antibiotic de-escalation was found. Future studies about the optimal cost-effective set of microbiological testing for antimicrobial stewardship in pneumonia patients should acknowledge the interaction between testing, way of communication of results and triggered physician alert systems.

**Trial registration:**

ClinicalTrials.gov NCT01743755.

## Background

Community-acquired pneumonia (CAP) is a frequent cause of hospital admission in the Western world [1]. Total costs for inpatient CAP care are estimated to be approximately €2,5 billion per year in Europe alone [2]. A previous Dutch multicenter study reported that average hospitalization cost were €4000 per patient admitted with CAP [3]. More recently, another Dutch multicenter study in adults > 65 years calculated average costs that were almost twice as high [4]. The majority of costs in both studies were nursing costs, which are directly related to length of hospital stay [3, 4].

Concepts like ‘Choosing Wisely’ target the best patient outcomes with optimal use of resources [5]. Because length of stay is an outcome, in other words the consequence of a multifactorial process, it cannot be modified nor standardized directly. In contrast, utilization of diagnostic tools and biomarkers, being the second largest cost driver in CAP care [3], potentially could be. Because narrowing antibiotic treatment when possible is considered an important outcome from an antimicrobial stewardship perspective, the present study aimed to systematically assess inter-hospital variation in the costs of diagnostics in the management of CAP in relation to antibiotic use.

## Methods

Data of adult patients hospitalized with CAP, who participated in a multicenter placebo-controlled trial investigating dexamethasone as adjunctive treatment were studied [6]. The present cohort (*n* = 300) is the mid-trial population. Patients were enrolled between October 2012 and October 2016 in four large Dutch teaching hospitals (Catharina Hospital Eindhoven, Canisius-Wilhelmina Hospital Nijmegen, Onze Lieve Vrouwe Gasthuis Amsterdam and the St. Antonius Hospital Nieuwegein/Utrecht). In short, the trial included immunocompetent patients aged ≥18 years, who were hospitalized with radiologically confirmed CAP, and who did not receive systemic corticosteroids at or shortly before admission and did not require direct intensive care unit (ICU) admission. The study protocol of the original investigation requested participating hospitals to perform pathogen identification including (but not restricted to) sputum cultures, blood cultures and urinary antigen testing (*Legionella pneumophila* serogroup 1 and *Streptococcus pneumoniae*). The study was approved by the Medical Ethical Committee of the St. Antonius Hospital and all patients provided written informed consent.

Hospital administration systems of the participating hospitals were consulted to determine resource utilization regarding diagnostics (laboratory testing and radiology) on a patient level. All care items billed to the individual patient’s health care insurance were captured. First, the captured items were grouped into three categories: 1) clinical chemistry testing, 2) radiological exams, and 3) microbiological testing. Second, only items from the period between admission and hospital discharge or ICU admission were retained. Next, for every hospital and every category, items were ranked based on mean number utilized per patient. After this step, we retained up to 5 items per category and only items that were utilized more than 0.1x on average per patient in one or more of the hospitals. Lastly, based on the identified top 5 items per category, average costs were calculated per category and per hospital. Standard item list prices from the Dutch Healthcare Authority (edition 2016) were used for all cost calculations [7].

In order to be able to link possible variations in resource use to relevant outcomes we captured the level of antibiotic de-escalation by day 3 of hospital stay. To assess antibiotic de-escalation at day 3, information on all prescribed antibiotics from day 1 to 3 was obtained from the Santeon Farmadatabase [8]. Whether antibiotics were de-escalated was determined per patient, where de-escalation was defined as narrowing of spectrum, switch to a different class of antibiotics, or switch from dual therapy to monotherapy. During the period in which patients were enrolled, the Dutch national guideline on the management of CAP advised an empirical antibiotic treatment based on the severity of disease. The antimicrobial spectrum varied from penicillin for mild pneumonia to a cephalosporin plus atypical coverage for severe CAP [9].

Besides antibiotic de-escalation, level of pathogen identification and secondary ICU admission rate were assessed. The latter as proxy for a complicated course of disease possibly caused by an insufficient diagnostic work-up. Descriptive statistics were used providing numbers (%), means (standard deviation (SD)) or medians [range] where appropriate. Categorical data were compared applying the Chi-square test. Differences in costs were analyzed with the Mann-Whitney U test.

## Results

### Patient characteristics

Mean age of the 300 studied patients was 64 years and 41% of the participants had a high PSI class (4–5). Both age and PSI class were significantly higher in hospital 4, as was the proportion of patients with heart failure and impaired renal function. This, together with the low number of patients from hospital 4 (*n* = 9 of 300), led to the decision to exclude hospital 4 from further analyses. Of the 291 remaining patients, the number with a PSI class of 4–5 at 45% was higher in hospital 1, compared to 30 and 28% in hospital 2 and 3, respectively. Other patient characteristics, including comorbidities and prior outpatient antibiotic treatment were similar in the hospitals.

### Resource utilization

Overall, 39% of all diagnostic items were ordered on day of admission and 59% up until day 2. Table 1 shows the items most frequently utilized per category per hospital. The mean amount of radiological exams performed was similar between hospitals. The number of clinical chemistry items was the lowest in hospital 1. In contrast, microbiological testing costs were highest in hospital 1, mainly due to performing the most polymerase chain reaction (PCR) hybridization tests (pathogen level). Microbiological testing was responsible for the majority of costs in every hospital. The mean difference in costs of microbiological testing was €531 (*p* < 0.001) per patient between hospital 1 and 2. Overall, the average costs per patient (€841) were highest in hospital 1 and lowest in hospital 2 (€350), *p* < 0.001.

**Table 1:** Ranking of mean number of items and mean costs per patient plus outcomes

Resource use	Hospital 1 (*n* = 201)	Hospital 2 (*n* = 50)	Hospital 3 (*n* = 40)
Clinical chemistry testing	Creatinine 2.90; 3 [0–22]	Creatinine 4.62; 5 [0–13]	Glucose 5.83; 2 [0–55]
	Potassium 2.73; 2 [0–48]	CRP 3.62; 3 [0–8]	Creatinine 4.95; 4 [0–12]
	Sodium 2.72; 2 [0–54]	Glucose 3.48; 2 [0–29]	CRP 4.90; 5 [0–11]
	CRP 2.21; 2 [0–13]	Hemoglobin 3.46; 3 [0–13]	Sodium 4.35; 4 [0–13]
	Leucocytes 1.92; 2 [0–16]	Sodium 3.32; 3 [0–12]	Potassium 4.35; 4 [0–11]
Mean costs (SE)	€25 (1.5)	€37 (2.9)	€49 (4.5)
Radiological exams	CXR 1.49; 1 [1–8]	CXR 1.40; 1 [0–6]	CXR 1.33; 1 [0–4]
	Chest CT 0.11; 0 [0–1]	Echocardiography 0.24; 0 [0–3]	Chest CT 0.13; 0 [0–1]
	Abdominal sonography 0.07; 0 [0–1]	Chest CT 0.16; 0 [0–1]	Abdominal sonography 0.13; 0 [0–1]
	Echocardiography 0.02; 0 [0–2]	Abdominal sonography 0.04; 0 [0–1]	Echocardiography 0.08; 0 [0–1]
Mean costs (SE)	€89 (7.5)	€118 (20)	€87 (17)
Microbiological testing	PCR – hybridization* 10.06; 9 [0–37]	*AMR detection* 3.66; *0 [0–57]*	PCR – hybridization* 5.33; 6 [0–15]
	Antigen detection 2.22; 2 [0–6]	Antigen detection 2.52; 2 [0–5]	Serologic antibody detection 4.25 5 [0–7]
	*AMR detection* 1.9; *0 [0–32]*	Blood culturing 2.40; 2 [0–6]	Blood culturing 2.2; 2 [0–6]
	Agar culturing 1.66; 1 [0–9]	Agar culturing 2.18; 2 [0–10]	*AMR detection* 1.93; *0 [0–44]*
	Gram staining 1.41; 1 [0–8]	Gram staining 1.6; 1 [0–5]	Antigen detection 1.58; 2 [0–4]
Mean costs (SE)	€726 (34)	€195 (17)	€476 (44)
Overall mean costs (SE)	€841 (37)	€350 (31)	€612 (46)
Outcomes	Hospital 1	Hospital 2	Hospital 3
Microbiological diagnostic yield, *n* (%)^$^	91 (45)	15 (30)	15 (38)
Antibiotic de-escalation, *n* (%)^**#**^	57 (30)	21 (48)	13 (33)
Secondary ICU admissions, *n* (%)	10 (5)	3 (6)	1 (3)

A maximum of 5 items per category per hospital are listed. Items are only listed if the mean number per patient was ≥0.1 in at least one hospital. Overall mean costs per patient per hospital per category are shown. *counted on pathogen level. Data are presented as mean; median [minimum-maximum], unless stated otherwise

*Abbreviations*: *AMR* antimicrobial resistance, *CRP* C-reactive protein, *CT* Computed Tomography, *CXR* Chest X-ray, *ICU* Intensive Care unit, *PCR* Polymerase Chain Reaction, *SE* Standard Error

^$^*p* = 0.050 for difference between hospital 1 and hospital 2

^#^*p* = 0.018 for difference between hospital 1 and hospital 2

### Outcomes

The level of pathogen identification varied from 45% in hospital 1 to 30% in hospital 2 (*p* = 0.050 comparing hospital 1 and hospital 2, see Table 1). Regarding antibiotic de-escalation, the de-escalation rate was highest in hospital 2 (48%), compared to 30 and 33% in hospital 1 and 3, respectively (*p* = 0.018 for difference between hospital 1 and 2). The most frequent de-escalations were from a penicillin plus atypical coverage to penicillin monotherapy (*n* = 20), from a cephalosporin plus atypical coverage to cephalosporin monotherapy (*n* = 17) and from a cephalosporin plus atypical coverage to atypical coverage only (*n* = 11). De-escalation numbers were calculated from 278 patients because at day 3 eight patients had been admitted to the ICU and five patients had been discharged. Percentages of patients receiving empirical dual therapy also varied between hospitals (1: 38%, 2: 30%, 3: 53%). Within the patients with dual therapy, the de-escalation rates were 51, 100, and 62% respectively. Overall, secondary ICU admission rates were 5, 6 and 3% in hospital 1, 2 and 3, respectively.

## Discussion

We showed large inter-hospital variations in utilization of diagnostics for in-hospital management of CAP between large Dutch teaching hospitals, mainly in microbiological testing. Considering an annual rate of 35,000 CAP-related hospital admission in the Netherlands [10], this suggests that considerable cost savings could be achieved when the most cost-effective panel of diagnostic tests could be identified.

In studies in pediatric patients with CAP, large inter-hospital variations in diagnostic testing have already been demonstrated [11, 12]. The present study now extends this finding to the management of adults with CAP. The observed large variation not only provides food for thought about cost-effective diagnostic work-up, but also shows that calculations of costs from single center settings need to be interpreted with caution because of limited external validity.

Based on our findings the variation in microbiological testing looks like a good candidate to start identification of the optimal set of diagnostics (e.g. PCR testing was ordered sporadically in hospital 2, whereas, in hospitals 1 and 3 it was ordered frequently). One would expect that the extent of microbiological testing would increase the chances of pathogen identification (which was the case in this study) and as such that there would be a positive association with de-escalation of antibiotic therapy. In the present study, comparing hospitals, however, an inverse association between testing costs and antibiotic de-escalation was observed. Discussions between the hospitals about this finding identified the fact that in hospital 2 an automated advisory message was sent to the treating physicians, alerting them as to when parenteral antibiotics had been administered for > 48 h and that clinical and pharmacological data did not preclude a switch from intravenous to oral therapy [13]. It was apparent that such an intervention along with microbiological investigation increased the likelihood that antibiotics would be de-escalated. In fact no patients in hospital 2 were still on dual therapy on day 3 probably because of earlier notification of microbiological results. In this approach physicians are actively directed to negative test results as well, in some cases also warranting antibiotic de-escalation (e.g. a negative *L. pneumophila* urinary antigen test [9]). This is in contrast to settings where only positive microbiological findings are actively communicated by the clinical microbiologist to the treating physician. We believe that further studies on optimal arrays of microbiological testing in CAP should take into account the potential interaction by automated trigger alerts, optionally present in many modern electronic health records. The approach of the present study can also be seen as an illustrative example for initiatives aiming to reduce health care costs. Our observed large variation in resource utilization and related costs led to discussions with unexpected novel insights eventually.

A limitation of our study is that the number of patients was relatively small and consisted of patients that participated in a clinical trial. Consequently, this could mean that the results may not be generalizable to all patients admitted with CAP. Nevertheless, this phenomenon applied equally to all hospitals and is therefore less likely to be the reason for the observed large inter-hospital variation. Second, in the present study, we chose to focus on variations in those tests that were most frequently utilized. This could imply an underestimation of the total variation in costs. On the other hand, these commonly utilized tests would be the main cost drivers and are likely to have the most impact if taken into account when altering the content of clinical protocols. Third, costs were calculated using price listing of the Dutch Health care Authority for 2016 (maximum prices), while true costs per item will be different and differ per hospital (due to specific agreements made between individual hospitals and health insurers). Lastly, antibiotic de-escalation was scored based on antibiotic prescription-based criteria solely. However, this should not have biased our results because the same criteria were applied for each hospital.

## Conclusions

Large inter-hospital variation exists with regard to diagnostic testing in the management of adult patients with community-acquired pneumonia. We found a counterintuitive inverse association between the costs for microbiological testing and antibiotic de-escalation rates, most likely due to an interactive electronic trigger tool designed to optimise intravenous to oral antibiotic switch in one of the hospitals. Future studies investigating the most cost-effective sets of microbiological testing that would optimize antimicrobial stewardship programs specific to patients admitted with CAP, should acknowledge the interaction between testing, and ways of communicating results to physicians.

## Abbreviations

CAP: Community-acquired pneumonia
ICU: Intensive care unit
PCR: Polymerase chain reaction
SD: Standard deviation
